# Serum metabolomics identified specific lipid compounds which may serve as markers of disease progression in patients with Alström and Bardet-Biedl syndromes

**DOI:** 10.3389/fmolb.2023.1251905

**Published:** 2023-11-06

**Authors:** Krzysztof Jeziorny, Karolina Pietrowska, Julia Sieminska, Ewa Zmyslowska-Polakowska, Adam Kretowski, Michal Ciborowski, Agnieszka Zmyslowska

**Affiliations:** ^1^ Department of Endocrinology and Metabolic Diseases, Polish Mother’s Memorial Hospital–Research Institute, Lodz, Poland; ^2^ Department of Paediatric Endocrinology, Medical University of Lodz, Lodz, Poland; ^3^ Clinical Research Centre, Medical University of Bialystok, Bialystok, Poland; ^4^ Department of Endodontics, Medical University of Lodz, Lodz, Poland; ^5^ Department of Clinical Genetics, Medical University of Lodz, Lodz, Poland

**Keywords:** metabolomic profiling, ALMS, BBS, ciliopathy, obesity, phospholipids

## Abstract

**Objectives:** Alström syndrome (ALMS) and Bardet-Biedl syndrome (BBS) are among the so-called ciliopathies and are associated with the development of multiple systemic abnormalities, including early childhood obesity and progressive neurodegeneration. Given the progressive deterioration of patients’ quality of life, in the absence of defined causal treatment, it seems reasonable to identify the metabolic background of these diseases and search for their progression markers. The aim of this study was to find metabolites characteristic to ALMS and BBS, correlating with clinical course parameters, and related to the diseases progression.

**Methods:** Untargeted metabolomics of serum samples obtained from ALMS and BBS patients (study group; n = 21) and obese/healthy participants (control group; each of 35 participants; n = 70) was performed using LC-QTOF-MS method at the study onset and after 4 years of follow-up.

**Results:** Significant differences in such metabolites as valine, acylcarnitines, sphingomyelins, phosphatidylethanolamines, phosphatidylcholines, as well as lysophosphatidylethanolamines and lysophosphatidylcholines were observed when the study group was compared to both control groups. After a follow-up of the study group, mainly changes in the levels of lysophospholipids and phospholipids (including oxidized phospholipids) were noted. In addition, in case of ALMS/BBS patients, correlations were observed between selected phospholipids and glucose metabolism parameters. We also found correlations of several LPEs with patients’ age (*p* < 0.05), but the level of only one of them (hexacosanoic acid) correlated negatively with age in the ALMS/BBS group, but positively in the other groups.

**Conclusion:** Patients with ALMS/BBS have altered lipid metabolism compared to controls or obese subjects. As the disease progresses, they show elevated levels of lipid oxidation products, which may suggest increased oxidative stress. Selected lipid metabolites may be considered as potential markers of progression of ALMS and BBS syndromes.

## Introduction

Alström syndrome (ALMS) and Bardet-Biedl syndrome (BBS) are rare genetic diseases classified as ciliopathies, in which the observed abnormalities are caused by dysfunction of primary cilia found in most cell types and involve in many important body processes, such as signal transduction, cell migration and regulation of the cell cycle (Marshall et al., 2015; Álvarez-Satta et al., 2017). These syndromes have an autosomal recessive mechanism of inheritance, and although a defect in only one gene occurs in ALMS (Marshall et al., 2011; Marshall et al., 2015) and causative variants affect more than 20 genes in patients with BBS (Katsanis et al., 2001; Forsythe et al., 2018), both syndromes have a very similar clinical presentation. Common major criteria include early-childhood obesity and retinal degeneration progressing to blindness. In addition, patients with BBS and ALMS often have additional conditions, such as type 2 diabetes mellitus (T2DM), insulin resistance, endocrinopathies (hypothyroidism, hypogonadism), hearing impairment, renal, cardiovascular or liver dysfunction and skeletal abnormalities. Intellectual disability in patients with BBS and motor development and learning problems in patients with ALMS are also identified (Marshall et al., 2011; Forsythe and Beales, 2013; Priya et al., 2016; Forsythe et al., 2018; Tahani et al., 2020; Jeziorny et al., 2022). Recent studies on the ciliary localization of leptin and melanocortin-4 receptors have confirmed that cilia play a crucial role in the transmission of metabolic signals in hypothalamic neurons, also indicating the central nature of the disorders observed in patients with ALMS and BBS (Davenport et al., 2007; Vaisse et al., 2017; Siljee et al., 2018). To date, no precise therapy has been found to inhibit the development of all components of both syndromes, and treatment is only symptomatic or targeted at particular disorders, including those of a central origin (Haws et al., 2020; Haws et al., 2021).

Due to the progressive nature of the observed symptoms, leading to shortened life expectancy of patients, it seems reasonable to look for markers of progression of ALMS and BBS syndromes. One of the promising methods may be metabolomics. Metabolomics is a relatively new technology that enables the evaluation of metabolites–small molecules formed during metabolism, the precise identification of which provides insight into biological processes. This allows a better understanding of cellular mechanisms and links them to the phenotype of patients. Metabolomics is gaining popularity and there are more and more attempts to demonstrate its usefulness in understanding the pathophysiology of diseases, especially rare diseases (Zmyslowska et al., 2017; Zacchia et al., 2020).

The aim of the study was to identify serum metabolites characteristic to ALMS and BBS and to correlate the identified compounds with clinical parameters and diseases progression.

## Material and methods

### Patients

The study protocol was approved by the University Bioethics Committee at the Medical University in Lodz, Poland (RNN/343/17/KE and RNN/267/18/KE). Patients and/or their parents gave written informed consent for participation in the study.

The study group comprised of 21 patients (aged 2–29 years at the first time-point) with genetically confirmed ALMS or BBS syndromes, as described previously (Zmyslowska et al., 2016; Jeziorny et al., 2020), including 13 ALMS patients with two mutations in the *ALMS* gene and eight BBS patients having at least two mutations in one of the six genes (*BBS2, BBS6, BBS7, BBS8, BBS9, BBS10*) under the care of the Rare diseases and Diabetogenetics Outpatient Clinic of the Department of Clinical Genetics in Lodz, Medical University of Lodz, Poland. The control groups included age-, sex- (*p* = 0.69) and BMI-matched (*p* = 0.4) overweight/obese (OB) subjects (n = 35) and age-matched healthy participants with normal body weight (Ctrl) (n = 35) and no metabolic disorders (*p* = 0.998). Table 1 shows characteristics of the study groups.

**TABLE 1:** Clinical characteristics of the study groups.

Comparison between BBS and ALMS group					
	BBS Me (IQR)		ALMS Me (IQR)		p
Age (years)	8.8 (10.9)		14 (8.5)		0.17
BMI (kg/m^2^)	28.1 (5.0)		30.6 (8.2)		0.28
At the beginning of the study					
	Study group - ALMS/BBS Me (IQR)	Patients with obesity - OB Me (IQR)		Healthy controls - Ctrl Me (IQR)	p
Age (years)	13.6 (11.4)	13.1 (6.3)		13.4 (10.8)	0.998
BMI (kg/m^2^)	28.6 (7.7)	29.6 (8.4)		-	0.4
Sex (F/M)	33%/67%	34%/66%		43%/57%	0.69
Comparison between time points (T1 vs T2)					
Age (years)	T1—13.6 (10.5)	N/A		N/A	0.0001
	T2—18.3 (12.9)				
BMI (kg/m^2^)	T1—29.8 (8.0)	N/A		N/A	0.2
	T2—30.8 (6.5)				

### Methods

Untargeted metabolomic analysis was performed on 105 serum samples obtained from AMLS and BBS (study group) and obese (OB)/healthy (Ctrl) groups after overnight fasting using LC-QTOF-MS method. Additionally, serum samples of 14 ALMS and BBS patients were also analyzed after 4-year follow-up (average of 4.64 years: 1.95-5.49; second time-point; T2) and were compared with those at study entry (first time-point; T1). The metabolomic profiling results were then compared with selected laboratory parameters, such as glucose metabolism parameters (HOMA-IR - Homeostatic Model Assessment for Insulin Resistance, glycated hemoglobin HbA1c and C-peptide levels) and renal function markers (serum creatinine and cystatin C).

### Serum metabolic fingerprinting

Metabolomic analysis was performed by liquid chromatography coupled to mass spectrometry (LC-MS) and tandem mass spectrometry (MS/MS), as described previously (Daniluk et al., 2019). Details are available in the Supplementary File.

### Statistical analysis

Multivariate statistics were used to evaluate data quality by checking the location of the QC samples on principal component analysis (PCA) plots and to observe sample discrimination on partial least squares discriminant analysis (PLS-DA) plots. Multivariate calculations and plots were performed by using SIMCA−P + 13.0.3.0 (Umetrics, Umea, Sweden).

One-way ANOVA with Tukey HSD *post hoc* test or paired non-parametric Mann-Whitney U test (for paired comparisons) were used to select statistically significant metabolic features. The obtained *p* values were corrected by Benjamini–Hochberg false discovery rate (FDR). The level of statistical significance was set at 95% (*p* < 0.05). Univariate statistics were performed using Mass Profiler Professional 15.1 software (Agilent, Santa Clara, CA, United States). Spearman rank correlation analyses were performed in MATLAB (R2015a) (MathWorks Inc., Natick, MA, United States).

One-way ANOVA, nonparametric Mann Whitney U test or Wilcoxon rank-sum test were used to calculate differences in age and BMI of study participants. These calculations were performed in GraphPad Prism 9.5.1 software (GraphPad Software, Boston, MA, United States). GraphPad Prism was also used to prepare box plots.

## Results

**FIGURE 1:**
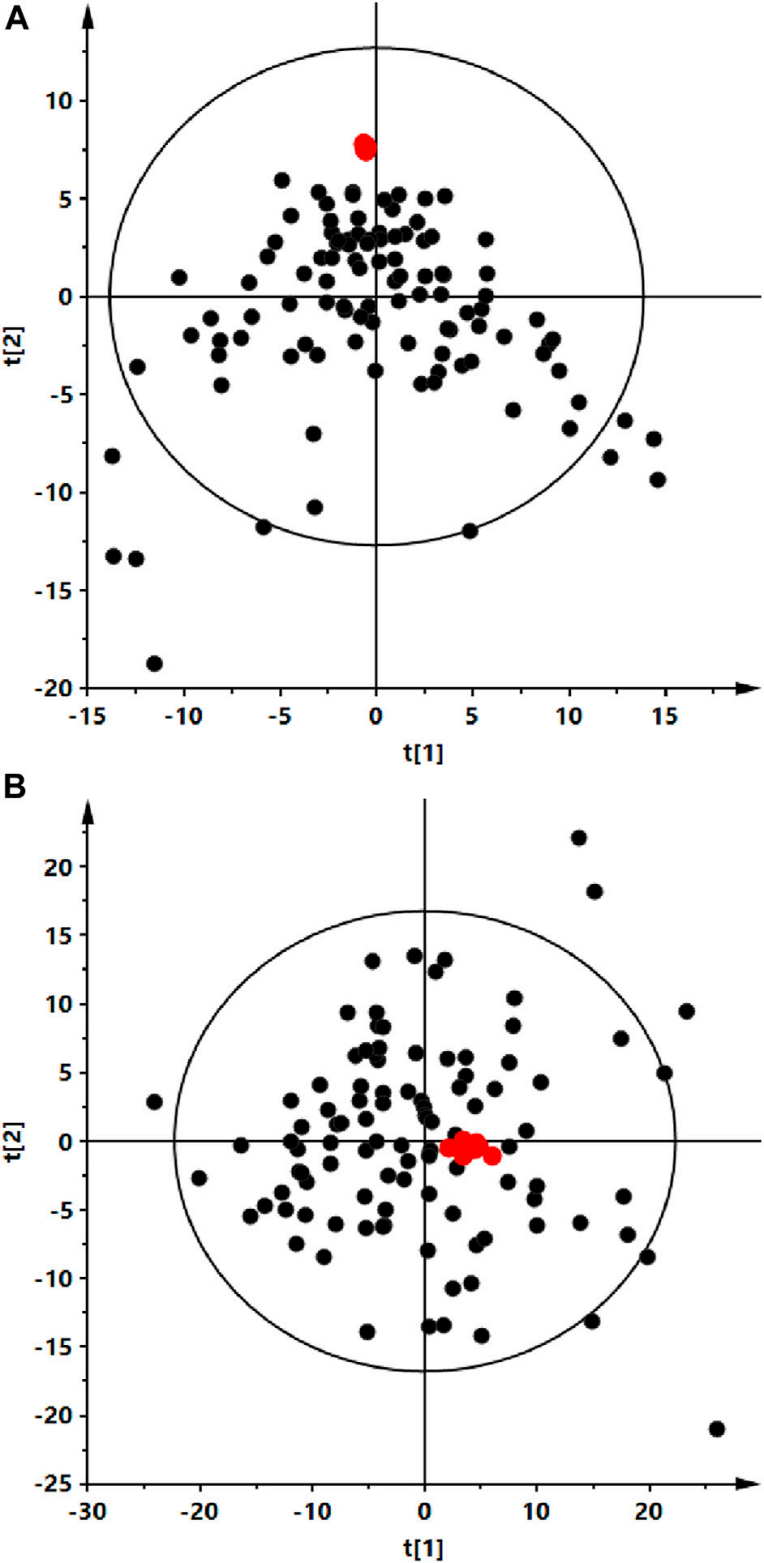
Principal component analysis (PCA) score plots of the serum fingerprints obtained for analyzed serum (black dots) and QC (red dots) samples. Panel **(A)** positive ion mode, R2 = 0.259. Panel **(B)** negative ion mode, R2 = 0.697.

**FIGURE 2:**
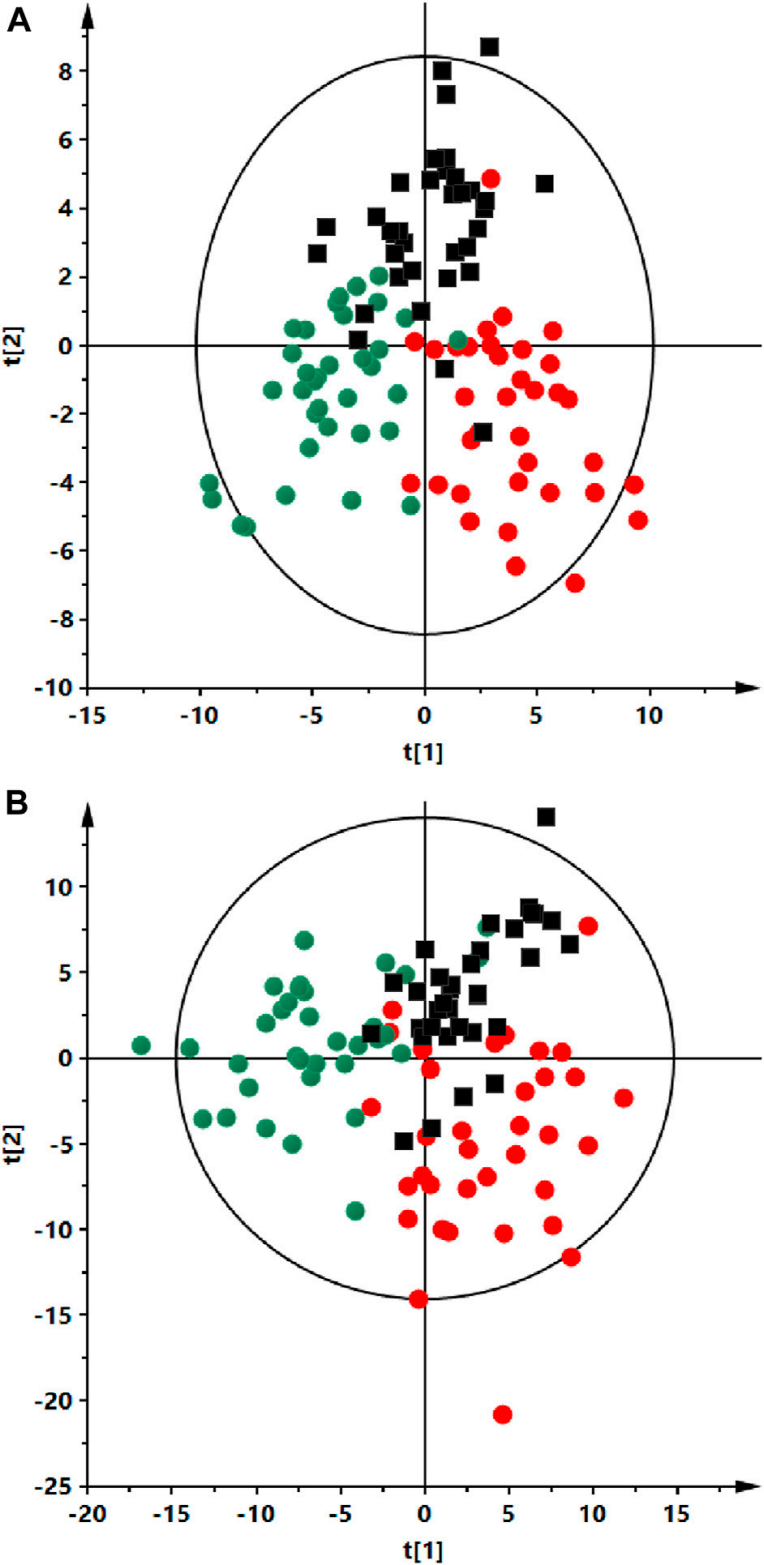
Partial least squares discriminant analysis (PLS-DA) score plots of the serum fingerprints of ALMS/BBS patients (red dots), obese participant (black squares) and a control group (green dots). Panel **(A)** positive ion mode, R2 = 0.735, Q2 = 0.412). Panel **(B)** negative ion mode, R2 = 0.734, Q2 = 0.048).

Analyses were performed using the LC-QTOF-MS method in both, positive and negative ion modes. Quality control of metabolomics data resulted in the selection of 393 reproducibly measured metabolic traits from the positive and 454 from the negative ion mode. In addition, the clustering of QC samples in the PCA plot is shown, which also confirms the reproducibility of the analyses performed (Figure 1). Figure 2 shows the clustering of samples according to the study groups: ALMS/BBS, obese patients (OB) and healthy controls (Ctrl). A series of statistical analyses were then performed for the following comparisons.

 I) ALMS vs. BBS,
 II) ALMS/BBS vs. Ctrl vs. OB,
 III) AMS/BBS T1 vs. T2,
 IV) ALMS/BBS vs. Ctrl vs. OB at first time-point,
 V) ALMS/BBS vs. Ctrl vs. OB at second time-point.

For the first comparison, no statistically significant differences were observed between the ALMS and BBS patients subgroups (*p* > 0.05). Therefore, despite the different genetic conditions underlying the two genetic syndromes, taking into account the common mechanism associated with damage to primary ciliary structures in ALMS and BBS syndromes, resulting in similar clinical manifestations in patients, as well as the convergence in the degree of obesity as measured by the BMI index and the lack of statistical differences in the age of the subjects and the results of metabolomic analyses obtained in the two study subgroups, for the purposes of this study, the two subgroups were combined (ALMS/BBS study group) to increase statistical power for these very rare genetic syndromes.

**FIGURE 3:**
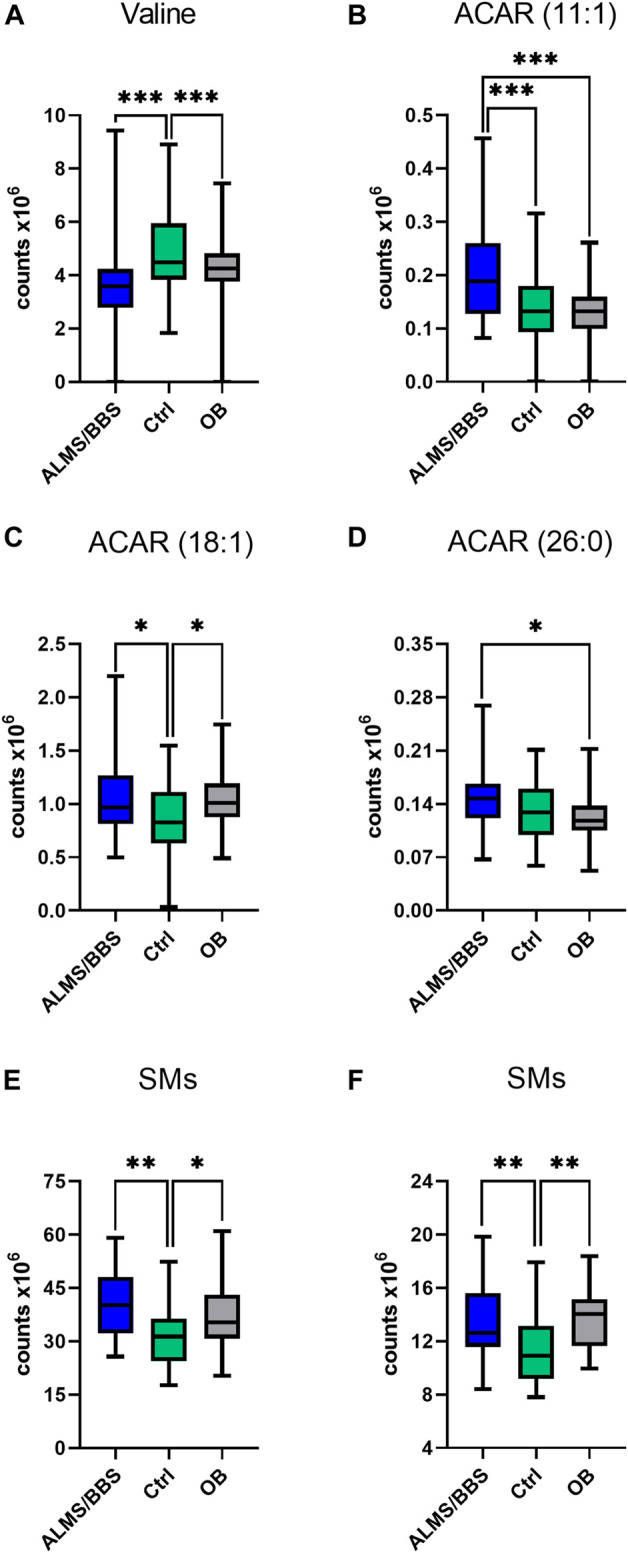
Comparison of selected metabolites in Alström/Bardet-Biedl patients (ALMS/BBS) vs Control groups (Ctrl) vs Obese participants (OB). Asterisks show *p*-value. *- *p* < 0.05, **- *p* < 0.01, ***- *p* < 0.001 **(A)** Valine **(B)** acylcarnitine 11:1 **(C)** acylcarnitine 18:1 **(D)** hexacosanoyl carnitine **(E)** SM (34:2), SM (36:2) **(F)** SM (36:1), SM (d18:2/14:0)

**FIGURE 4:**
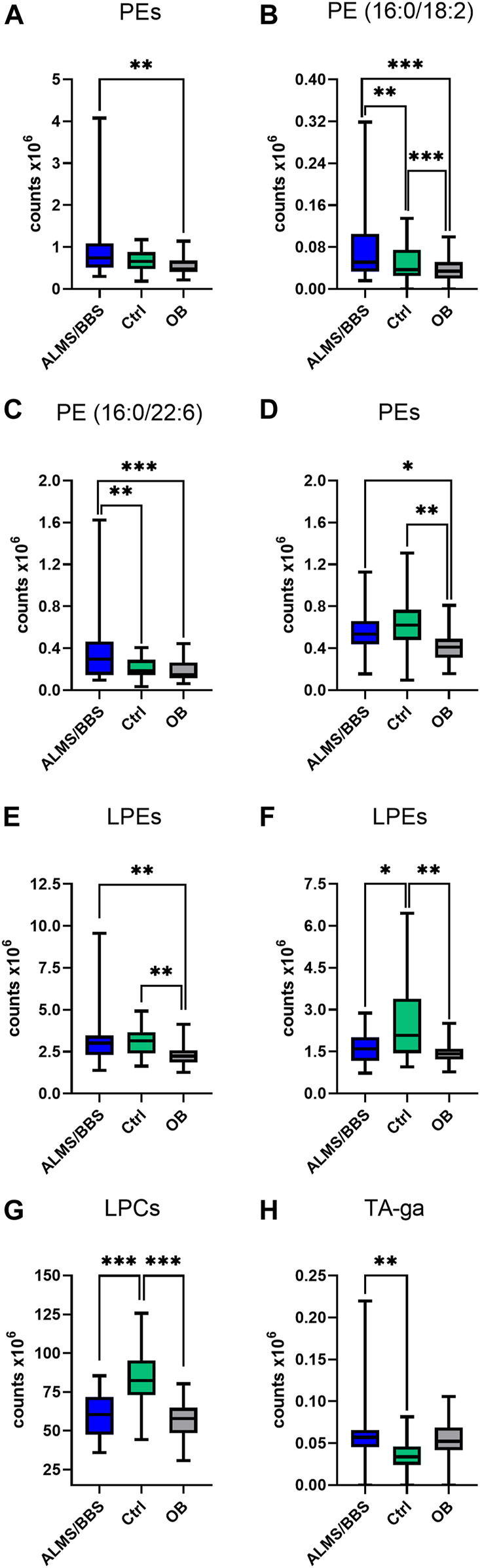
Comparison of selected metabolites in Alström/Bardet-Biedl patients (ALMS/BBS) vs Control groups (Ctrl) vs Obese participants (OB). Asterisks show *p*-value. *- *p* < 0.05, **- *p* < 0.01, ***- *p* < 0.001 **(A)** PE (36:4), PE (16:0/20:4), PE (20:5/P-16:0)/PE (20:5/O-16:1) **(B)** PE (16:0/18:2) **(C)** PE (16:0/22:6) **(D)** PE (18:2/18:1), PE (18:2/P-16:0)/PE (18:2/O-16:1), PE (O-34:3)/PE (P-34:2) **(E)** LPE (16:0) sn-1, LPE (16:0) sn-2, LPE (18:0) sn-1, LPE (18:0) sn-2, LPE (18:1), LPE (16:0) sn-1, LPE (16:0) sn-2, LPE (18:0) sn-1, LPE (O-18:1)/LPE (P-18:0) **(F)** LPE (18:2) sn-1, LPE (18:2) sn-2, LPE (18:2) sn-1, LPE (18:2) sn-2, LPE (22:5), LPE (18:0) sn-2 **(G)** LPC (18:1) sn-1, LPC (18:1) sn-2, LPC (18:2) sn-1, LPC (18:2) sn-2, LPC (19:0), LPC (20:0), LPC (20:1) sn-1, LPC (20:1) sn-2, LPC (20:2), LPC (O-18:1)/LPC (P-18:O), LPC (17:1) sn-1, LPC (17:1) sn-2 **(H)** tetrahydroaldosterone-3-glucuronide.

**FIGURE 5:**
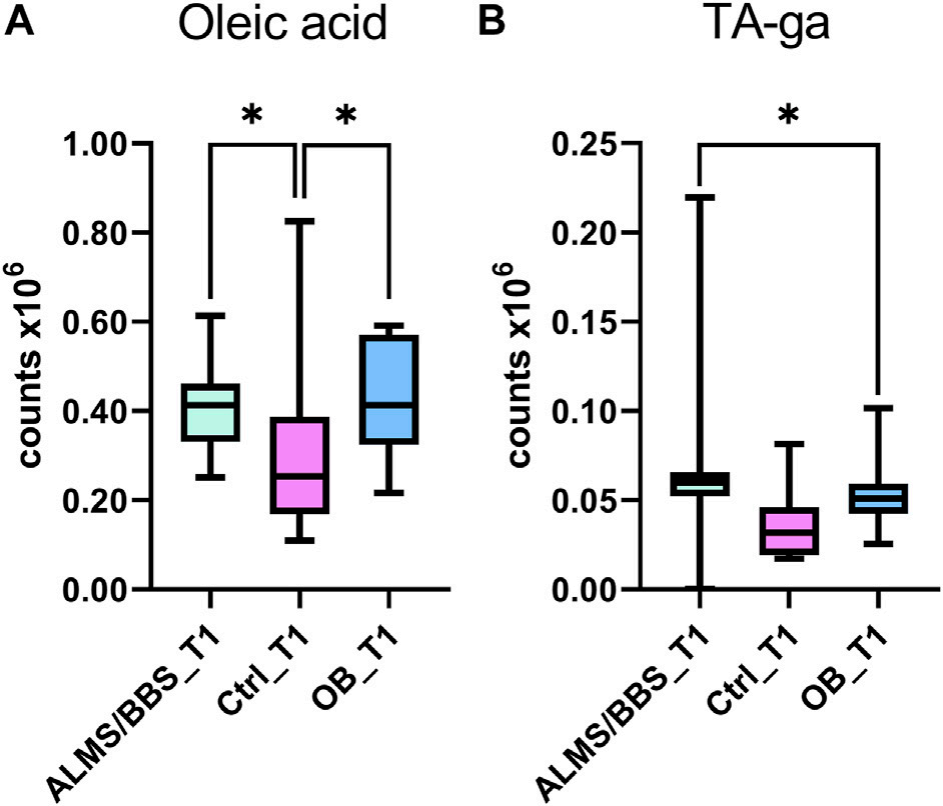
Comparison of selected metabolites in Alström/Bardet-Biedl patients (ALMS/BBS) vs Control groups (Ctrl) vs Obese participants (OB) at first time-point (T1). Asterisks show *p*-value. *- *p* < 0.05 **(A)** oleic acid **(B)** tetrahydroaldosterone-3-glucuronide.

Based on the results obtained for ALMS/BBS vs. Ctrl vs. OB comparison (Supplementary Table S1 in Supplementary file), we observed decreased level of valine (Figure 3A), as well as increased levels of acylcarnitines (Figures 3B–D), sphingomyelins (Figures 3E, F), and majority of phosphatidylethanolamines (PE) (Figures 4A–D), as well as decreased levels of lysophosphatidylethanolamines (LPE) (Figures 4E, F) and lysophosphatidylcholines (LPC) (Figure 4G) and elevated tetrahydroaldosterone-3-glucuronide level (Figure 4H) in ALMS/BBS patients compared to controls. LPC and LPE had the highest levels in control subjects and were at similar levels in OB and ALMS/BBS patients. Comparing ALMS/BBS vs. Ctrl vs. OB at study entry (T1) (Supplementary Table S1), mainly phospholipids (PL) were found statistically significant, which changes were similar to those observed in ALMS/BBS vs. Ctrl vs. OB comparison. Also, changes in the levels of oleic acid and tetrahydroaldosterone-3-glucuronide were noted, which had the lowest levels in the control group and were at similar levels in the other groups (ALMS/BBS and OB) (Figures 5A, B).

**FIGURE 6:**
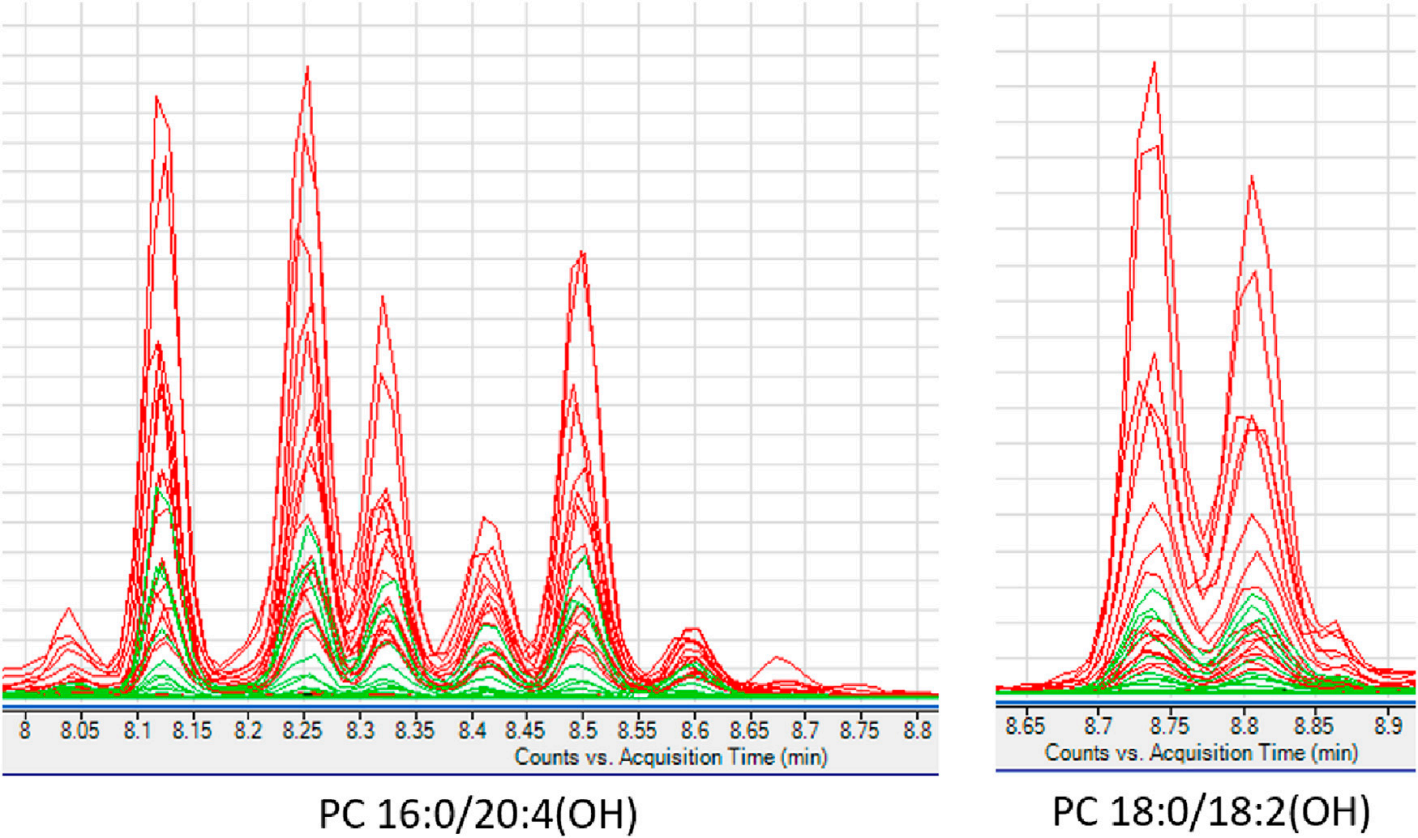
Extracted ion chromatograms of oxidized phosphatidylcholines detected in studied serum samples. ALMS/BBS–red, Control group–black, Obese participants–green.

**FIGURE 7:**
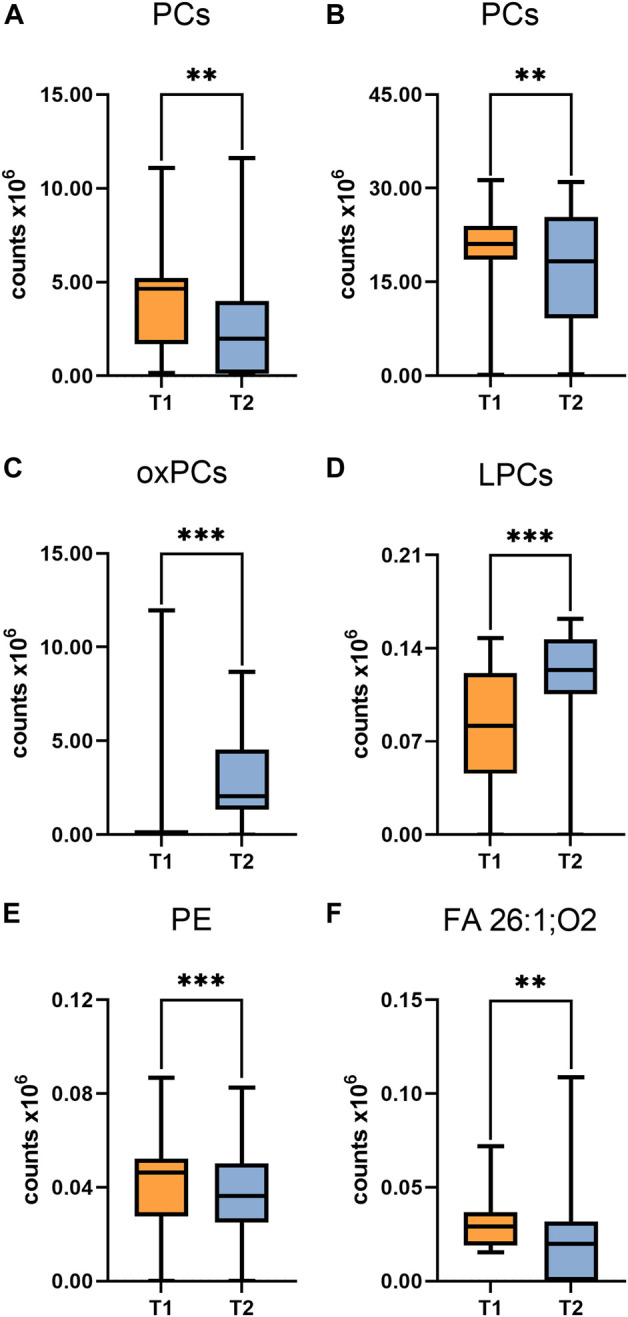
Comparison of selected metabolites in Alström/Bardet-Biedl group at first time-point (T1) vs Second time-point. Asterisks show *p*-value. **- *p* < 0.01, ***- *p* < 0.001 **(A)** PC (30:1), PC (34:3) **(B)** PC (36:4), PC (38:4) **(C)** PC (16:0/20:4(OH)), PC (16:0/20:4(OH)), PC (18:0/18:2(OH)) **(D)** LPC (20:1) sn-2, LPC (20:3) **(E)** PE (20:5/P-16:0)/PE (20:5/O-16:1) **(F)** hexacosanedioic acid (FA 26:1; O2).

Interesting results were obtained in an analogous comparison for the second time point (T2) (Supplementary Table S3 in Supplementary file). Again, similar changes were observed in the levels of the same PL and lysophospholipids (LPL) groups as in the previous comparison (T1), and in addition, elevated levels of oxidized PC (oxPC), i.e., PC 16:0/20:4(OH) and PC 18:0/18:2(OH), were observed in ALMS/BBS patients (Figure 6). These compounds were also found in some samples from obese individuals and were below measurable levels in the other study participants. Curiously, these compounds were detected in only two patients in the ALMS/BBS group at T1, while they were not detected in only one patient at T2. Moreover, comparing the compounds in the study group between the two time points, mainly changes in the levels of LPL and lysophospholipids, including oxPC were observed. Levels of LPC (Figure 7D) and oxPC (Figure 7C) were elevated at T2 compared to T1, while levels of PC (Figures 7A, B), PE (Figure 7E) and FA (Figure 7F) were decreased.

Then, analyzing the results of the identified compounds from metabolomics to routine laboratory parameters of the clinical course in patients with ALMS and BBS, strong correlations between selected phospholipids and parameters of glucose metabolism such as: HbA1c (indicator of chronic hyperglycaemia), HOMA-IR (marker of insulin resistance) and C-peptide levels (marker of insulin secretion) were observed (Table 2). Significant correlations were also observed between levels of some LPE, PC and acylcarnitines and serum creatinine values of patients with ALMS and BBS. Detailed statistically significant correlations between metabolomics and clinical parameters are shown in Table 2 (*p* < 0.05).

**TABLE 2:** Correlation between compounds identified by metabolomic analysis and clinical parameters in the study group.

Compound	Clinical parameter	r	p
LPC(20:0)	HOMA	−0.6967	0.0056
LPC(22:6)	HOMA	0.6176	0.0186
LPC(O-16:1)/LPC(P-16:0)	HOMA	−0.7846	0.0009
LPC(O-18:0)	HOMA	−0.8198	0.0003
LPC(O-18:1)/LPC(P-18:0)	HOMA	−0.6703	0.0087
LPC(O-18:2)/LPC(P-18:1)	HOMA	−0.5824	0.0289
LPE(22:5)	HOMA	−0.7846	0.0009
LPE(22:6)	HOMA	0.6044	0.0221
LPE(22:6)	HOMA	0.5692	0.0336
PC(16:0/22:6)	HOMA	0.7011	0.0052
PC(18:2/18:2)	HOMA	−0.5604	0.0371
PE(16:0/22:6)	HOMA	0.6835	0.0070
PE(16:0/20:4)	HOMA	0.5736	0.0320
ST19:1; O3; S	HOMA	0.6615	0.0100
Leucine/Isoleucine	HOMA	0.5692	0.0336
CAR (18:1)	HOMA	0.6308	0.0156
LPC (O-16:1)/LPC (P-16:0)	HOMA	−0.7582	0.0017
LPC (O-16:0)	HOMA	−0.7802	0.0010
LPC (O-18:1)/LPC (P-18:O)	HOMA	−0.6659	0.0093
LPC (O-18:0)	HOMA	−0.8462	0.0001
LPE (22:6)	HOMA	0.6088	0.0209
LPC (19:0)	HOMA	−0.5385	0.0470
PE (36:4)	HOMA	0.5473	0.0428
PC (O-34:2)/PC (P-34:1)	HOMA	−0.5648	0.0353
PC (18:0/18:2(OH))	HOMA	−0.8330	0.0002
PC (38:5)	HOMA	0.6264	0.0165
PC (40:6)	HOMA	0.7187	0.0038
LPC(O-18:0)	Hba1c	−0.5491	0.0420
LPC(O-18:2)/LPC(P-18:1)	Hba1c	−0.6461	0.0126
LPE(18:0)	Hba1c	0.5976	0.0240
LPE(22:5)	Hba1c	−0.6924	0.0061
PE(16:0/20:4)	Hba1c	0.5667	0.0346
SM(d32:2)	Hba1c	−0.6770	0.0078
trans-2-Dodecenoylcarnitine	Hba1c	0.5954	0.0247
CAR (18:1)	Hba1c	0.5380	0.0472
LPC (O-16:0)	Hba1c	−0.5821	0.0289
Ceramide (34:1)	Hba1c	0.5601	0.0372
SM (d18:2/14:0)	Hba1c	−0.7409	0.0024
PE (36:4)	Hba1c	0.6174	0.0186
PC (O-34:2)/PC (P-34:1)	Hba1c	−0.6659	0.0093
PC (18:0/18:2(OH))	Hba1c	−0.5402	0.0461
LPC(19:0)	C-peptide	−0.5560	0.0389
LPC(20:0)	C-peptide	−0.5912	0.0260
LPC(O-16:1)/LPC(P-16:0)	C-peptide	−0.8198	0.0003
LPC(O-18:0)	C-peptide	−0.8462	0.0001
LPC(O-18:1)/LPC(P-18:0)	C-peptide	−0.5780	0.0304
LPC(O-18:2)/LPC(P-18:1)	C-peptide	−0.5341	0.0492
LPE(22:5)	C-peptide	−0.7143	0.0041
PC(18:2/20:4)	C-peptide	−0.5473	0.0428
PE(16:0/22:6)	C-peptide	0.5868	0.0274
ST19:1; O3; S	C-peptide	0.7011	0.0052
LPC (O-16:1)/LPC (P-16:0)	C-peptide	−0.7670	0.0014
LPC (O-16:0)	C-peptide	−0.6615	0.0100
LPC (O-18:1)/LPC (P-18:O)	C-peptide	−0.7099	0.0045
LPC (O-18:0)	C-peptide	−0.8593	0.0001
LPC (19:0)	C-peptide	−0.5692	0.0336
LPC (24:0)	C-peptide	−0.5473	0.0428
PC (18:0/18:2(OH))	C-peptide	−0.7582	0.0017
PC (40:6)	C-peptide	0.6747	0.0081
LPC(22:6)	creatinine	0.5516	0.0408
LPE(18:0)	creatinine	0.7363	0.0027
LPE(20:4)	creatinine	0.6044	0.0221
LPE(22:6)	creatinine	0.7538	0.0018
LPE(22:6)	creatinine	0.7582	0.0017
PC(16:0/20:5)	creatinine	0.5560	0.0389
PC(16:0/22:6)	creatinine	0.6835	0.0070
PE(16:0/18:2)	creatinine	0.5692	0.0336
PE(16:0/22:6)	creatinine	0.6088	0.0209
Leucine/Isoleucine	creatinine	0.5604	0.0371
CAR (18:1)	creatinine	0.8813	0.0000
LPE (22:6)	creatinine	0.7846	0.0009
Ceramide (34:1)	creatinine	0.5385	0.0470
Hexacosanoyl carnitine	creatinine	0.5516	0.0408
PC (18:0/18:2(OH))	creatinine	−0.5648	0.0353
PC (20:5/16:0)	creatinine	−0.5385	0.0470
PC (40:6)	creatinine	0.5341	0.0492
LPC(18:0)	creatinine	0.5516	0.0408
LPC(20:1)	creatinine	0.6879	0.0065
LPC(O-18:0)	creatinine	0.6044	0.0221
PC(16:0/20:4)	creatinine	−0.6264	0.0165
Valine	creatinine	−0.6484	0.0121
Linoleamide	creatinine	−0.6396	0.0138
LPE (18:2) sn-2	creatinine	0.6044	0.0221
Hexacosanoyl carnitine	creatinine	0.6088	0.0209
LPC (20:1) sn-2	creatinine	0.6000	0.0233
LPC (20:1) sn-1	creatinine	0.7011	0.0052

**FIGURE 8:**
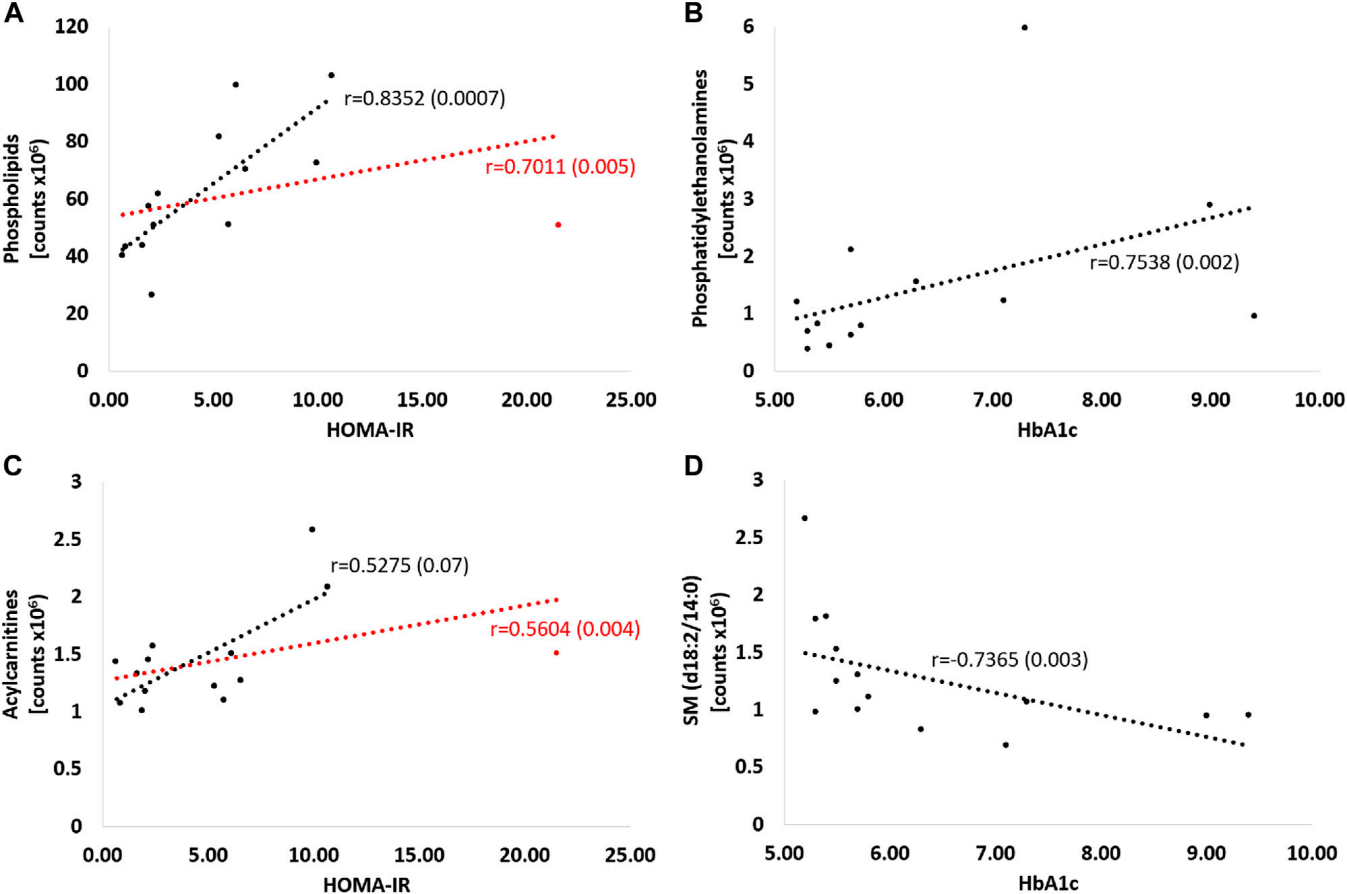
Correlations of selected metabolites with parameters of glucose metabolism - HOMA-IR and Hba1c. Red curve: all patients; black curve: after exclusion of a patient with an outlier HOMA-IR result. Detailed description in the text of the manuscript, *p*-values in brackets. **(A)** PC (16:0/20:4), PC (16:0/20:5), PC (16:0/22:6), PC (18:2/18:2), PC (18:2/20:4), PC (O-34:2) or PC (P-34:1), PC (38:5), PC (20:5/16:0), PC (40:6), PC (18:0/18:2(OH)), PE (16:0/18:2), PE (16:0/22:6), PE (16:0/20:4) **(B)** PE (16:0/18:2), PE(16:0/22:6), PE(16:0/20:4) **(C)** dodecenoylcarnitine, octadecenoylcarnitine, hexacosanoylcarnitine **(D)** SM (d18:2/14:0).

Figure 8 shows strong positive correlations between HOMA-IR and both phospholipids (Figure 8A) and acylcarnitines (Figure 8C), including all patients (respectively: r = 0.70; *p* = 0.005 and r = 0.56; *p* = 0.004), and excluding one participant in the study group with an outlier HOMA-IR value (21.52) (r = 0.83; *p* = 0.0007 and r = 0.53; *p* = 0.07, respectively). Also HbA1c value correlated positively with PE (r = 0.75, *p* = 0.002; Figure 8B) and negatively with SM (d18:2/14:0) (r = −0.74, *p* = 0.003; Figure 8D).

**FIGURE 9:**
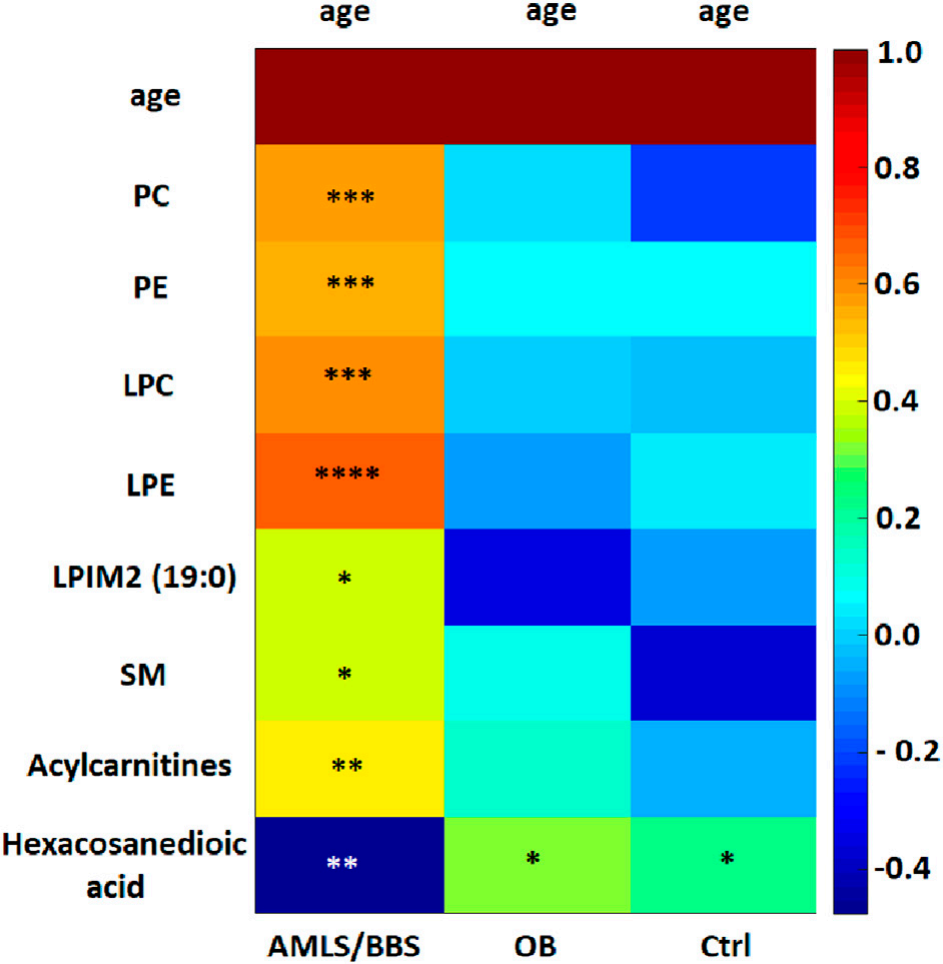
Heatmap of correlations of selected metabolites with age in Alström/Bardet-Biedl study group (ALMS/BBS), control group (Ctrl) and obese group (OB). Asterisks show *p*-value. *- *p* < 0.05, **- *p* < 0.01, ***- *p* < 0.001, ****- *p* < 0.00001 PC: PC (36:5), PC (38:5), PC (40:6), PC (40:6), PC (16:0/22:6), PC (20:5/P-16:0) or PC (20:5/O-16:1), PC (36:5). PE: PE (16:0/22:6), PE (36:4) LPC: LPC (20:3), LPC (22:6), LPC (O-18:0), LPC (20:5) sn-2, LPC (20:5) sn-1, LPC (20:0) LPE: LPE (18:0), LPE (20:4), LPE (20:5), LPE (22:6) LPIM2 (19:0) - nonadecanoylglycero-phospho-myo-inositol SM: SM (34:2), SM (d18:0/16:1(OH)) Acylcarnitines: Dodecenoylcarnitine, Hexacosanoylcarnitine

**FIGURE 10:**
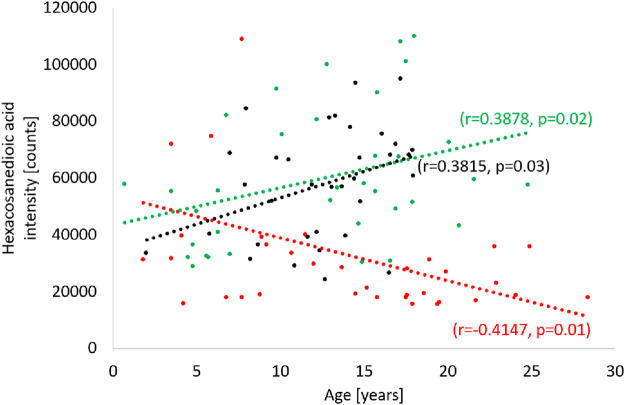
Correlation of hexacosanedioic acid with age in Alström/Bardet-Biedl study group (red), control group (green) and obese group (black).

Analyzing the results obtained for the ALMS/BBS study group in terms of identifying markers of disease progression, correlations of several metabolites with the age of the patients were noted (*p* < 0.05) (Figure 9). These are mainly different types of lipids, primarily LPE. Other identified metabolites in this correlation analysis are: hexacosanoylcarnitine, LPC (20:5), LPC (22:6), LPE (22:6), LPE (20:4), LPE (22:6), LPE (22:6), PC (38:5), PC (40:6), PC (16:0/22:6), PC (36:5), PE (36:4), PE (16:0/22:6), SM (d18:0/16:1(OH)). Interestingly, levels of only one metabolite (hexacosanedioic acid) correlated negatively with age of the ALMS and BBS patients, while positively in the other groups (Figure 10).

## Discussion

Our study is the first to evaluate the utility of metabolomics in patients with Alström syndrome and one of the few to examine its use in patients with Bardet-Biedl syndrome. Metabolomics is quite new examination method and seems to be encouraging ones to determine potential markers of disease progression. Up to date there are no direct studies which revealed specific metabolites for both ALMS and BBS syndromes. Since the main common symptom of Alström and Bardet-Biedl syndromes is early childhood obesity, one can look for possible differences between the observed metabolite concentrations in patients with the syndromes and non-syndromic obesity, resulting mainly from hyperphagia.

In our patients with ALMS/BBS syndromes higher levels of acylcarnitines were observed. Similarly, increased levels of acylcarnitines, but in urine samples were noted in patients with metabolic syndrome by Yu Zhi-rui et al. (Yu et al., 2014). Several studies revealed also higher acylcarnitines levels in association with insulin resistance in patients with obesity or T2DM (Mihalik et al., 2010; Schooneman et al., 2013; Arjmand et al., 2022; Shi et al., 2023). In addition, Arjmand et al. found in non-diabetic patients negative correlation between HOMA-IR and CAR 18:1 and strong positive between leucine and HOMA-IR, as we observed in our study (Arjmand et al., 2022). In the study of Mihalik et al. higher levels of long-chain acylcarnitines in obese and T2DM patients were also observed (Mihalik et al., 2010). On the other hand, Shooneman et al. noted no correlation between acylcarnitine levels and clinical indicators of glucose metabolism, doubting their involvement in such disturbances as insulin resistance and type 2 diabetes mellitus (Schooneman et al., 2016). According to Marchese et al. high molecular weight lipids from the acylcarnitine and phosphatidylcholine classes were associated with a decrease in e-GFR leading to renal failure (Marchese et al., 2022). We also observed that certain types of LPE and PC positively correlated with serum creatinine levels in ALMS/BBS patients. In our study, both the ALMS/BBS and OB groups had significantly higher levels of tetrahydroaldosterone-3-glucuronide, a metabolite of aldosterone, than non-obese subjects. Similar observations and its positive correlation with obesity and hypertension were reported by Zhi-rui et al. and Marks et al. (Marks et al., 1985; Yu et al., 2014).

Several studies have shown an association of metabolites with patients’ BMI and obesity (Fernandez et al., 2013; Ho et al., 2016; Carayol et al., 2017; Libert et al., 2018; Auguet et al., 2023). In our study, we obtained consistent results with the observations of Ho et al. These authors found a negative correlation between BMI and LPC and LPE, and a positive correlation between BMI and SM (Ho et al., 2016). Significant reductions in the concentrations of some LPCs were also observed by Wang et al. in groups of obese subjects and diabetic obese patients compared to non-obese individuals (Wang et al., 2019).

We further observed that patients with ALMS/BBS and obese patients had relatively reduced LPC levels and elevated SM levels compared to the non-obese group. Several studies have shown an association of LPC and SM with chronic diseases. Those with significant coronary calcification showed reduced levels of various LPCs, in contrast to those without calcification (Sakamoto et al., 2017). Lower LPC levels have also been found in patients with neurodegenerative diseases like Alzheimer’s disease (Lin et al., 2017; Otoki et al., 2023). Moreover, it is thought that the onset of cardiovascular disease may be preceded by decreased levels of certain LPCs (LPC 16∶0 and LPC 20∶4) and increased levels of specific SM (SM 38:2) (Fernandez et al., 2013). Some recent studies have also shown that changes in sphingolipid metabolism may also be related to the development of micro- and macrovascular complications of diabetes (Klein et al., 2014; Lopes-Virella et al., 2019; Sojo et al., 2023).

In our study, ALMS/BBS patients were also characterized by higher levels of oxidized PCs, which are identified as markers of oxidative stress. Interestingly, novel studies by Dong et al. and Xue et al. have pointed to the potential involvement of oxidized PCs in the development of neurodegenerative diseases, including multiple sclerosis (Dong et al., 2021; Xue et al., 2023).

Many studies have consistently indicated that long-chain fatty acids have the ability to affect carbohydrate, lipid and protein metabolism, and contribute to the onset and progression of metabolic syndrome caused by insulin resistance. In our study, long-chain fatty acid (FA 26:1; O2) was the only metabolite in the study group that correlated negatively with age in the study group, while positively in both control groups. To date, this metabolite has not been observed to be associated with obesity, insulin resistance or metabolic syndrome. However, a recent study of a long-chain fatty acid similar to FA26:1, but with four methyl groups attached to the chain (3,3,14,14-tetramethylhexadecanedioic acid) confirmed its hypolipemic and antidiabetic properties, as well as its modulating effect on insulin secretion (Mayorek et al., 1997; Las et al., 2006; Kalderon et al., 2012). Further studies are needed to confirm the similar properties of FA26:1; O2 and to decide whether it can be considered a marker of ALMS/BBS progression.

Several limitations of our study should be mentioned. Due to the rarity of Alström and Bardet-Biedl syndrome in the European population, only a limited number of ALMS/BBS patients was included and combined into one study group. However, it should be emphasized that metabolic studies were conducted at two, distant time points. Another limitation may be the lack of results of routine laboratory tests in control subjects and lipids profile evaluation in study participants. In addition, the metabolites assessed in this study represent only a small portion of the human metabolome. It may be necessary to further expand the range of features studied in order to discover a specific marker of disease progression.

## Conclusion

Concluding, our metabolomics study have shown that patients with ALMS/BBS have altered lipid metabolism compared to controls or obese individuals, although their metabolic profile is partially similar to obese individuals. As the disease progresses, elevated levels of lipid oxidation products have been noted, which may indicate increased oxidative stress. This may suggest abnormalities in lipid metabolism, energy metabolism (acylcarnitine) and oxidative stress in the course of ciliopathies. Moreover, hexacosanedioic acid seems to be a promising marker of disease progression in Alström and Bardet-Biedl syndromes. However, further studies and validation of its long-term utility are needed.

## Data Availability

The raw data supporting the conclusion of this article will be made available by the authors, without undue reservation.

## Abbreviations

ALMS, Alström syndrome; BBS, Bardet-Biedl syndrome; BMI, body mass index; CAR, acylcarnitin; eGFR, estimated glomerular filtration rate; FA, fatty acid; Hba1c, glycated haemoglobin; HOMA-IR, Model Assessment for Insulin Resistance; IQR, interquartile range; LC-QTOF-MS, liquid chromatography quadrupole time-of-flight mass spectrometry; LPC, lysophosphatidylcholine; LPE, lysophosphatidylethanolamine; LPIM2, nonadecanoylglycero-phospho-myo-inositol; LPL, lysophospholipid; Me, median; MS/MS, tandem mass spectrometry; PAPC, 1-palmitoyl-2-arachidonoyl-sn-glycero-3-phosphocholine; PC, phosphatidylcholine; PCA, principal component analysis; PE, phosphatidylethanolamine; PLS-DA, partial least squares discriminant analysis; SM, sphingomyelin; ST, sterol lipid; T1, first time-point; T2, second time-point; T2DM, type 2 diabetes mellitus.
