# Supplementary material for: Serum metabolomics identified specific lipid compounds which may serve as markers of disease progression in patients with Alström and Bardet-Biedl syndromes
Source: Front Mol Biosci. 2023 Nov 6;10:1251905. doi: 10.3389/fmolb.2023.1251905 (PMC10657895; doi:10.3389/fmolb.2023.1251905)
Supplement: Supplementary file 1 [file Table1.DOCX]

Supplementary Material to:

**SERUM METABOLOMICS IDENTIFIED SPECIFIC LIPID COMPOUNDS WHICH**

**MAY SERVE AS MARKERS OF DISEASE PROGRESSION IN PATIENTS**

**WITH ALSTRÖM AND BARDET-BIEDL SYNDROMES**

Krzysztof Jeziorny^1,2^, Karolina Pietrowska^3^, Julia Sieminska^3^, Ewa Zmyslowska-Polakowska^4^, Adam Kretowski^3^, Michal Ciborowski^3^, Agnieszka Zmyslowska^5^

^1^Department of Endocrinology and Metabolic Diseases, Polish Mother’s Memorial Hospital–Research Institute, 93-338 Lodz, Poland

^2^Department of Paediatric Endocrinology, Medical University of Lodz, 93-338 Lodz, Poland

^3^Clinical Research Centre, Medical University of Bialystok, Bialystok, Poland

^4^Department of Endodontics, Medical University of Lodz, 93-338 Lodz, Poland

^5^Department of Clinical Genetics, Medical University of Lodz, 92-213 Lodz, Poland

**Corresponding author:**

Prof. Agnieszka Zmysłowska MD, PhD.

Department of Clinical Genetics, Medical University of Lodz, Poland

Pomorska Str. 251, 92-213, Lodz, Poland, tel./fax +48 42 272 57 67

e-mail: [agnieszka.zmyslowska@umed.lodz.pl](mailto:agnieszka.zmyslowska@umed.lodz.pl)

# Serum metabolic fingerprinting

*Chemicals and reagents*

Purified water was obtained using the Milli-Q Integral 3 system (Millipore SAS, Molsheim, France). Zomepirac sodium salt (used as the internal standard, IS), oleic acid, linoleamide, lysophosphatidylethanolamine (LPE) 18:0, phosphatidylethanolamine (PE) 16:0/22:6, LC-MS-grade acetonitrile, methanol, formic acid, and LC-grade ethanol were purchased from Sigma-Aldrich Chemie GmbH (Steinheim, Germany). A mixture of oxidized PAPCs (oxPAPCs) was purchased from Avanti (Avanti Polar Lipids, Inc. AL, USA).The API-TOF reference mass solution kit (G1969-850001) and tuning solutions, ESI-L low-concentration tuning mix (G1969-85000), and ESI-TOF Biopolymer Analysis reference masses (G1969-850003) were purchased from Agilent Technologies (Santa Clara, CA, USA).

*Sample treatment and analysis*

Serum samples were prepared by simultaneous protein precipitation and metabolite extraction performed by vortex-mixing (for 1 minute) of one serum volume with four volumes of freezing cold (–20°C) methanol/ethanol (1:1) mixture containing 1 ppm zomepirac. After extraction, samples were stored on ice for 10 minutes and centrifuged at 21,000×g for 20 minutes at 4°C. The supernatant was filtered through a 0.22-µm nylon filter into glass vials. Quality control (QC) samples were prepared by mixing an equal volumes of all samples. Obtained mixture was prepared following the same procedure as the rest of the samples.

Samples were analyzed in the randomized order by a liquid chromatography coupled with mass spectrometry (LC-MS) system consisting of 1290 Infinity LC with a degasser, 2 binary pumps, and a thermostated autosampler coupled to a 6545 Q-TOF-MS detector (both Agilent Technologies, Santa Clara, CA, USA). Analyses were performed in positive (ESI+) and negative (ESI-) ion modes, whereby 1 µL of sample was injected into a thermostated (60°C) Zorbax Extend- C18 RRHT (2.1×50 mm, 1.8-µm particle size, Agilent Technologies) chromatographic column. The flow rate was 0.6 mL/min with solvent A (water with 0.1% formic acid) and solvent B (acetonitrile with 0.1% formic acid). The chromatographic gradient started at 5% of phase B for the first minute, followed by an increase of phase B to 80% (from 1 to 7 minutes) and to 100% (from 7 to 11.5 minutes). After reaching 100%, the gradient returned to initial conditions (5% phase B) in 0.5 minutes, which were kept from 12 to 15 minutes. The mass spectrometer was operated in full scan mode from mass (m/z) 50–1000. The capillary voltage was set to 3 kV for positive and 4 kV for negative ionization mode. Nozzle voltage was 1000 V. The drying gas flow rate was 12 L/min at 250°C and gas nebulizer at 52 psig; the fragmentor voltage was 175 V for positive and 200V for negative ionization modes. Data were collected in centroid mode at a scan rate of 1.5 scan per second. Accurate mass measurements were obtained by means of calibrant solution delivery using a dual-nebulizer ESI source. A calibrating solution containing reference masses at m/z 121.0509 (protonated purine) and m/z 922.0098 (protonated hexakis [1H,1H,3H-tetrafluoropropoxy] phosphazine or HP-921) in positive ion mode or m/z 119.0363 (proton abstracted purine) and m/z 966.0007 (formate adduct of HP-921) in negative ion mode was continuously introduced by an isocratic pump (Agilent, Santa Clara, CA, USA) at a flow rate of 0.5 mL/min (1:100 split).

*LC-MS data treatment*

The raw data collected by the analytical instrumentation were cleaned of background noise and unrelated ions by the molecular feature extraction (MFE) tool in Mass Hunter Qualitative Analysis Software (B.07.00, Agilent, Santa Clara, CA, USA). The MFE creates a list of all possible components described by mass, retention time (RT), and abundance. The limit for the background noise for data extraction by MFE was set to 2500 and 1000 counts for positive and negative ion mode, respectively. To identify co-eluting adducts of the same feature, the following adduct settings were applied: +H, +Na, +K in positive ion mode and −H, +HCOO, +Cl for negative ion mode. Dehydration neutral losses were also allowed in both ionization modes. Sample alignment and data filtering were performed using Mass Profiler Professional 15.1 (Agilent, Santa Clara, CA, USA). Parameters applied for the alignment were 1% for RT and 15 ppm for the mass variation. In the quality assurance (QA) procedure, metabolic features detected in >80% in QC samples with the coefficient of variation (CV) <20% were kept for further data treatment.

*Metabolites identification*

Identification of metabolites was performed based on the tandem mass spectrometry (MS/MS) fragmentation, as previously described [1]. Accurate masses of features were searched against the METLIN, KEGG, LIPIDMAPS, and HMDB databases, which were simultaneously accessed by CEU Mass Mediator (http://ceumass.eps.uspceu.es/mediator/faces/index.xhtml) [2]. The identity of metabolites was confirmed by matching the experimental MS/MS spectra to MS/MS spectra from databases or fragmentation spectra and retention time obtained for the metabolite’s standard. Experiments were repeated with identical chromatographic conditions to the primary analysis. Ions were targeted for collision-induced dissociation (CID) fragmentation on the fly based on the previously determined accurate mass and retention time. Phospholipids and acylcarnitines with typical fragmentation patterns were identified based on previously described characteristic fragments [3, 4]. Oxidized phospholipids were identified based on the characteristic fragments observed in a mixture of oxidized PAPCs [5].

Table S1. Metabolites significant in comparison II.

| metabolite | p-value | | | | | ALMS/BBS | | Ctrl | | Obese | |
| --- | --- | --- | --- | --- | --- | --- | --- | --- | --- | --- | --- |
|  |  |  |  |  |  | Intensity [counts x 10^3^] | | | | | |
|  | [ALMS/BBS] vs [Ctrl] vs [Obese] | [ALMS/BBS] vs [Ctrl] vs [Obese]* | [Ctrl] vs [ALMS/BBS] | [Obese] vs [ALMS/BBS] | [Obese] vs [Ctrl] | median | IQR | median | IQR | median | IQR |
| valine | 0.003 | 0.01 | 0.0001 | ns | 0.0002 | 3588 | 1316 | 4491 | 2008 | 4264 | 1041 |
| CAR (11:1) | 0.0003 | 0.002 | 0.0001 | 0.0001 | ns | 189 | 125 | 132 | 84 | 132 | 58 |
| CAR (18:1) | 0.008 | 0.03 | 0.01 | ns | 0.03 | 967 | 429 | 829 | 457 | 1012 | 303 |
| Hexacosanoyl carnitine | 0.02 | 0.05 | ns | 0.01 | ns | 147 | 44 | 129 | 56 | 118 | 31 |
| FA 26:1;O2 | 0.00000005 | 0.000001 | 0.0001 | 0.0001 | ns | 23 | 195 | 55 | 32 | 60 | 31 |
| ST 21:1;O3;S | 0.02 | 0.04 | ns | 0.002 | 0.0001 | 190 | 137 | 172 | 238 | 271 | 216 |
| Tetrahydroaldosterone-3-glucuronide | 0.000002 | 0.00002 | 0.004 | ns | ns | 57 | 20 | 34 | 21 | 52 | 26 |
| LPC (16:0(OH)) | 0.02 | 0.05 | ns | 0.01 | ns | 209 | 113 | 187 | 79 | 148 | 91 |
| LPC (17:0) | 0.02 | 0.04 | 0.0004 | ns | 0.0001 | 660 | 531 | 797 | 165 | 558 | 198 |
| LPC (18:0) | 0.02 | 0.04 | ns | 0.05 | 0.01 | 118 | 40 | 127 | 14 | 109 | 22 |
| LPC(18:1) | 0.002 | 0.008 | 0.02 | 0.003 | 0.0001 | 28929 | 11181 | 36065 | 10064 | 24923 | 7726 |
| LPC(18:2) | 0.01 | 0.03 | 0.0001 | ns | 0.01 | 33415 | 12197 | 45758 | 13398 | 32148 | 7936 |
| LPC(18:3) | 0.009 | 0.02 | 0.009 | 0.003 | 0.001 | 278 | 222 | 330 | 392 | 225 | 95 |
| LPC(19:0) | 0.00001 | 0.0001 | 0.0001 | ns | 0.0001 | 95 | 80 | 135 | 27 | 90 | 51 |
| LPC(20:0) | 0.0000003 | 0.000005 | 0.0001 | ns | 0.0001 | 120 | 62 | 184 | 64 | 114 | 41 |
| LPC(20:1) | 0.0000001 | 0.000001 | 0.0001 | ns | 0.0001 | 355 | 140 | 509 | 102 | 335 | 94 |
| LPC(20:2) | 0.01 | 0.03 | 0.002 | 0.0001 | 0.0001 | 373 | 166 | 480 | 158 | 310 | 138 |
| LPC(20:3) | 0.006 | 0.02 | 0.0002 | 0.0001 | ns | 108 | 51 | 107 | 31 | 90 | 36 |
| LPC(20:5) | 0.02 | 0.05 | ns | 0.02 | 0.01 | 1228 | 740 | 1062 | 508 | 842 | 575 |
| LPC(22:4) | 0.006 | 0.02 | 0.01 | ns | 0.02 | 67 | 44 | 94 | 34 | 71 | 33 |
| LPC(22:5) | 0.00005 | 0.0003 | 0.0002 | ns | 0.002 | 160 | 84 | 261 | 121 | 176 | 99 |
| LPC(22:6) | 0.009 | 0.02 | ns | ns | 0.009 | 56 | 27 | 52 | 21 | 47 | 19 |
| LPC (O-16:0) | 0.004 | 0.02 | ns | 0.04 | 0.004 | 889 | 638 | 1048 | 334 | 749 | 237 |
| LPC(O-16:1)/LPC(P-16:0) | 0.0002 | 0.001 | 0.04 | ns | 0.0002 | 686 | 429 | 865 | 211 | 648 | 245 |
| LPC(O-18:0) | 0.002 | 0.009 | 0.0001 | ns | 0.001 | 246 | 213 | 314 | 115 | 223 | 59 |
| LPC(O-18:1)/LPC(P-18:0) | 0.0000003 | 0.000008 | 0.0001 | ns | 0.0001 | 87 | 74 | 140 | 40 | 82 | 31 |
| LPC(O-16:1)/LPC(P-16:0) | 0.0005 | 0.002 | ns | ns | 0.0004 | 166 | 108 | 206 | 47 | 158 | 49 |
| LPC(O-18:2)/LPC(P-18:1) | 0.002 | 0.006 | 0.006 | 0.0001 | 0.0001 | 22 | 17 | 26 | 7 | 17 | 21 |
| LPE(16:0) | 0.0002 | 0.002 | ns | 0.0009 | 0.0005 | 1103 | 475 | 1201 | 591 | 852 | 279 |
| LPE(18:0) | 0.02 | 0.04 | 0.003 | 0.003 | 0.003 | 1518 | 628 | 1577 | 705 | 1206 | 380 |
| LPE(18:1) | 0.000008 | 0.00007 | 0.05 | 0.02 | 0.0001 | 817 | 313 | 996 | 643 | 659 | 215 |
| LPE(18:2) | 0.004 | 0.02 | 0.04 | ns | 0.004 | 1491 | 777 | 1964 | 1783 | 1315 | 348 |
| LPE(20:4) | 0.01 | 0.03 | ns | ns | 0.01 | 127 | 50 | 133 | 57 | 114 | 21 |
| LPE(20:5) | 0.02 | 0.04 | ns | 0.0002 | ns | 28 | 17 | 29 | 23 | 19 | 26 |
| LPE(22:5) | 0.006 | 0.02 | ns | 0.02 | 0.01 | 197 | 130 | 219 | 60 | 175 | 27 |
| LPE(22:6) | 0.02 | 0.04 | ns | 0.01 | ns | 500 | 259 | 416 | 204 | 396 | 149 |
| LPE(P-18:0)/LPE(O-18:1) | 0.00005 | 0.0005 | ns | 0.001 | 0.0002 | 419 | 366 | 431 | 220 | 291 | 86 |
| LPE (P-16:0) | 0.005 | 0.02 | ns | 0.003 | ns | 214 | 186 | 204 | 87 | 156 | 60 |
| LPE(O-18:0) | 0.0007 | 0.003 | 0.02 | ns | ns | 21 | 18 | 23 | 8 | 20 | 5 |
| LPE(P-16:0)/LPE(O-16:1) | 0.0006 | 0.003 | ns | 0.0005 | ns | 151 | 116 | 132 | 76 | 113 | 36 |
| LPI(18:2) | 0.02 | 0.04 | ns | ns | 0.01 | 103 | 37 | 119 | 43 | 101 | 38 |
| LPIM2 (19:0) | 0.000004 | 0.00004 | ns | ns | ns | 19 | 26 | 12 | 16 | 17 | 9 |
| PC (20:5/16:0) | 0.009 | 0.03 | 0.03 | ns | 0.02 | 233 | 60 | 200 | 56 | 225 | 62 |
| PC (32:1) | 0.002 | 0.01 | 0.002 | ns | ns | 17234 | 15408 | 11819 | 5698 | 14824 | 7801 |
| PC (36:3) | 0.007 | 0.03 | 0.02 | ns | 0.02 | 109045 | 25986 | 106217 | 16856 | 108472 | 19440 |
| PC (36:5) | 0.0003 | 0.002 | 0.0003 | ns | ns | 26965 | 19993 | 17991 | 15131 | 20262 | 17805 |
| PC (38:5) | 0.02 | 0.05 | ns | ns | ns | 14255 | 7492 | 12490 | 4548 | 13145 | 5233 |
| PC (38:7) | 0.000001 | 0.00002 | 0.0001 | ns | 0.0001 | 82 | 68 | 137 | 59 | 87 | 49 |
| PC (38:8) | 0.009 | 0.03 | ns | 0.001 | 0.001 | 110 | 68 | 89 | 58 | 95 | 60 |
| PC (40:6) | 0.009 | 0.03 | ns | 0.005 | ns | 1950 | 1079 | 1621 | 527 | 1839 | 549 |
| PC (O-34:2)/PC (P-34:1) | 0.0007 | 0.004 | ns | 0.0001 | 0.0001 | 2491 | 1394 | 3489 | 1413 | 2222 | 981 |
| PC (O-38:5)/PC (P-38:4) | 0.01 | 0.03 | 0.01 | ns | 0.04 | 12045 | 4564 | 14370 | 3364 | 12425 | 5401 |
| PC (O-38:7)/PC (P-38:6) | 0.000002 | 0.00002 | ns | 0.005 | 0.0001 | 762 | 280 | 941 | 265 | 607 | 135 |
| PC(16:0/18:3) &PC(16:1/18:2) | 0.01 | 0.03 | ns | 0.02 | ns | 585 | 335 | 680 | 284 | 709 | 254 |
| PC(16:0/20:3) | 0.003 | 0.009 | 0.01 | ns | 0.006 | 2544 | 991 | 2184 | 563 | 2502 | 794 |
| PC(16:0/20:4) | 0.02 | 0.04 | 0.0001 | ns | 0.0001 | 4912 | 777 | 4585 | 740 | 4918 | 700 |
| PC(16:0/20:5) | 0.0002 | 0.001 | 0.0001 | ns | 0.0001 | 63 | 34 | 48 | 26 | 56 | 26 |
| PC(16:0/22:6) | 0.02 | 0.04 | 0.02 | ns | ns | 4104 | 1266 | 3433 | 1187 | 3856 | 675 |
| PC(16:1/16:0) | 0.002 | 0.006 | 0.002 | ns | 0.03 | 957 | 619 | 655 | 295 | 864 | 370 |
| PC(17:0/18:2) | 0.01 | 0.02 | ns | 0.01 | ns | 80 | 84 | 107 | 65 | 72 | 58 |
| PC(18:2/18:2) | 0.000006 | 0.00005 | 0.005 | 0.02 | ns | 1040 | 719 | 1603 | 446 | 1079 | 386 |
| PC(18:2/20:4) | 0.00002 | 0.0001 | 0.0001 | 0.0001 | ns | 320 | 172 | 475 | 153 | 385 | 82 |
| PC(18:3/18:2) | 0.0000003 | 0.000004 | 0.0001 | 0.0002 | 0.0002 | 45 | 27 | 77 | 44 | 49 | 21 |
| PC(20:4/P-18:0) /PC(20:4/O-18:1) | 0.009 | 0.02 | 0.007 | ns | ns | 410 | 135 | 481 | 87 | 440 | 75 |
| PC(20:5/P-16:0) /PC(20:5/O-16:1) | 0.003 | 0.009 | 0.0001 | ns | 0.0001 | 34 | 27 | 35 | 23 | 27 | 14 |
| PC(32:1) | 0.008 | 0.02 | 0.008 | ns | ns | 76 | 50 | 59 | 24 | 70 | 25 |
| PC(36:4) | 0.0000006 | 0.000007 | ns | ns | ns | 122 | 80 | 181 | 77 | 134 | 45 |
| PC(36:5) | 0.0001 | 0.0008 | ns | ns | ns | 51 | 26 | 36 | 23 | 43 | 22 |
| PE (36:4) | 0.003 | 0.01 | ns | 0.002 | ns | 639 | 478 | 527 | 338 | 404 | 223 |
| PE (O-34:3)/PE (P-34:2) | 0.004 | 0.02 | ns | 0.04 | 0.004 | 434 | 197 | 526 | 264 | 369 | 161 |
| PE(16:0/18:2) | 0.005 | 0.02 | 0.005 | 0.0001 | 0.0004 | 51 | 62 | 37 | 49 | 34 | 25 |
| PE(16:0/20:4) | 0.001 | 0.004 | ns | 0.0007 | ns | 91 | 76 | 75 | 50 | 58 | 33 |
| PE(16:0/22:6) | 0.0003 | 0.001 | 0.009 | 0.0003 | ns | 296 | 259 | 183 | 125 | 148 | 135 |
| PE(18:2/18:1) | 0.003 | 0.01 | ns | 0.0001 | 0.0001 | 21 | 21 | 22 | 30 | 12 | 18 |
| PE(18:2/P-16:0) /PE(18:2/O-16:1) | 0.00003 | 0.0002 | ns | 0.0001 | 0.0001 | 52 | 37 | 65 | 25 | 37 | 22 |
| PE(20:5/P-16:0) /PE(20:5/O-16:1) | 0.004 | 0.01 | ns | 0.0002 | ns | 24 | 21 | 17 | 33 | 11 | 19 |
| SM (34:2) | 0.0005 | 0.003 | 0.0004 | ns | 0.05 | 24715 | 8465 | 19242 | 8277 | 21614 | 6111 |
| SM (36:1) | 0.0003 | 0.002 | 0.01 | ns | 0.0004 | 11801 | 3345 | 10274 | 3635 | 13295 | 3265 |
| SM (36:2) | 0.0006 | 0.004 | 0.002 | ns | 0.003 | 15300 | 7278 | 10869 | 5730 | 14339 | 4969 |
| SM (d18:2/14:0) | 0.00000007 | 0.000003 | ns | 0.04 | ns | 302 | 168 | 183 | 115 | 295 | 123 |
| SM(d32:2) | 0.0004 | 0.002 | 0.0009 | ns | 0.004 | 174 | 63 | 126 | 56 | 178 | 50 |
| SM(d33:2) | 0.001 | 0.005 | 0.0001 | 0.0009 | ns | 22 | 9 | 15 | 15 | 20 | 9 |
| SM(d36:1) | 0.01 | 0.03 | ns | ns | 0.009 | 627 | 177 | 557 | 150 | 658 | 196 |
| Ceramide (34:1) | 0.004 | 0.02 | ns | 0.01 | 0.01 | 338 | 82 | 328 | 110 | 297 | 93 |

*- FDR corrected p-values.

Table S2. Metabolites significant in comparison IV.

| metabolite | p-value | | | | | ALMS/BBS | | Ctrl | | Obese | |
| --- | --- | --- | --- | --- | --- | --- | --- | --- | --- | --- | --- |
|  |  |  |  |  |  | Intensity [counts x 10^3^] | | | | | |
|  | [ALMS/BBS] vs [Ctrl] vs [Obese] | [ALMS/BBS] vs [Ctrl] vs [Obese]* | [Ctrl] vs [ALMS/BBS] | [Obese] vs [ALMS/BBS] | [Obese] vs [Ctrl] | median | IQR | median | IQR | median | IQR |
| Tetrahydroaldosterone-3-glucuronide | 0.0003 | 0.003 | ns | 0.05 | ns | 60 | 12 | 32 | 23 | 51 | 14 |
| FA 26:1;O2 | 0.00001 | 0.0003 | 0.0002 | 0.0002 | ns | 29 | 16 | 56 | 43 | 68 | 34 |
| Oleic acid | 0.007 | 0.04 | 0.03 | ns | 0.01 | 413 | 104 | 254 | 187 | 413 | 219 |
| LPC(16:1) | 0.008 | 0.03 | ns | ns | ns | 146 | 36 | 100 | 53 | 111 | 31 |
| LPC(18:1) | 0.0002 | 0.005 | 0.02 | ns | 0.0003 | 28453 | 4167 | 37313 | 9627 | 24783 | 6363 |
| LPC(18:2) | 0.008 | 0.03 | 0.02 | ns | 0.02 | 29141 | 12715 | 47746 | 13033 | 32901 | 7547 |
| LPC(18:3) | 0.008 | 0.03 | 0.05 | ns | 0.002 | 338 | 162 | 365 | 434 | 224 | 57 |
| LPC (19:0) | 0.002 | 0.01 | 0.007 | ns | 0.04 | 96 | 82 | 135 | 21 | 77 | 45 |
| LPC(20:0) | 0.0003 | 0.003 | 0.0003 | ns | 0.0002 | 121 | 71 | 195 | 52 | 112 | 34 |
| LPC(20:1) | 0.0003 | 0.006 | 0.0001 | 0.002 | 0.0004 | 299 | 129 | 504 | 98 | 334 | 120 |
| LPC(20:2) | 0.001 | 0.008 | 0.03 | ns | 0.0008 | 353 | 105 | 447 | 131 | 262 | 119 |
| LPC(20:3) | 0.0007 | 0.006 | ns | 0.0006 | 0.03 | 87 | 46 | 58 | 18 | 43 | 17 |
| LPC (22:5) | 0.007 | 0.03 | 0.02 | ns | 0.01 | 157 | 132 | 260 | 121 | 170 | 65 |
| LPC(O-18:1)/LPC(P-18:O) | 0.00003 | 0.0007 | 0.0002 | 0.02 | 0.04 | 76 | 57 | 141 | 25 | 76 | 25 |
| LPC(O-16:1)/LPC(P-16:0) | 0.002 | 0.01 | 0.02 | ns | 0.002 | 138 | 95 | 209 | 50 | 152 | 52 |
| LPE(16:0) | 0.0009 | 0.01 | ns | 0.008 | 0.001 | 1270 | 493 | 1237 | 447 | 845 | 238 |
| LPE(18:0) | 0.004 | 0.03 | ns | 0.02 | 0.006 | 1606 | 692 | 1577 | 456 | 1095 | 339 |
| LPE(18:1) | 0.00005 | 0.0009 | 0.02 | 0.02 | 0.0001 | 344 | 77 | 404 | 225 | 242 | 38 |
| LPE(18:2) | 0.0002 | 0.002 | 0.01 | ns | 0.0002 | 1567 | 505 | 2671 | 1710 | 1390 | 272 |
| LPE(P-18:0)/LPE(O-18:1) | 0.01 | 0.05 | ns | ns | 0.01 | 118 | 76 | 175 | 87 | 105 | 28 |
| PC (32:1) | 0.0007 | 0.01 | 0.0008 | 0.02 | ns | 27300 | 15937 | 11932 | 9166 | 13459 | 8371 |
| PC (36:3) | 0.0005 | 0.008 | 0.001 | ns | 0.003 | 34101 | 6430 | 40773 | 5315 | 34802 | 8104 |
| PC (36:5) | 0.006 | 0.04 | 0.005 | ns | ns | 28148 | 25740 | 15921 | 16304 | 19926 | 14321 |
| PC (38:7) | 0.0004 | 0.006 | ns | 0.0004 | 0.0001 | 78 | 50 | 127 | 37 | 66 | 34 |
| PC (40:6) | 0.002 | 0.02 | 0.002 | ns | ns | 2116 | 682 | 1477 | 365 | 1725 | 603 |
| PC (O-38:7)/PC (P-38:6) | 0.004 | 0.03 | ns | ns | 0.003 | 731 | 215 | 937 | 258 | 563 | 77 |
| PC(16:0/20:5) | 0.002 | 0.01 | 0.002 | ns | ns | 68 | 40 | 44 | 28 | 55 | 23 |
| PC(16:0/22:6) | 0.01 | 0.04 | 0.009 | ns | ns | 4201 | 1066 | 3237 | 964 | 3735 | 715 |
| PC(16:1/16:0) | 0.001 | 0.01 | 0.001 | 0.04 | ns | 1281 | 475 | 718 | 386 | 818 | 458 |
| PC(18:0/20:5) | 0.004 | 0.02 | 0.004 | 0.03 | ns | 626 | 377 | 430 | 132 | 473 | 218 |
| PC(18:2/20:4) | 0.01 | 0.05 | 0.0001 | 0.0002 | ns | 309 | 144 | 460 | 118 | 376 | 102 |
| PC(18:3/18:2) | 0.002 | 0.01 | 0.0001 | ns | 0.02 | 48 | 25 | 73 | 27 | 39 | 18 |
| PC(32:1) | 0.002 | 0.01 | 0.002 | ns | ns | 107 | 29 | 61 | 32 | 71 | 26 |
| PC(36:4) | 0.0001 | 0.002 | ns | 0.002 | 0.0001 | 128 | 45 | 184 | 46 | 119 | 44 |
| PC(36:5) | 0.01 | 0.04 | 0.008 | ns | ns | 52 | 20 | 33 | 26 | 42 | 18 |
| PE(16:0/20:4) | 0.01 | 0.04 | ns | 0.009 | ns | 98 | 66 | 85 | 55 | 65 | 39 |
| PE(16:0/22:6) | 0.009 | 0.03 | ns | 0.009 | ns | 301 | 332 | 183 | 98 | 148 | 132 |
| SM(d32:2) | 0.00001 | 0.0003 | 0.0001 | ns | 0.001 | 195 | 82 | 112 | 35 | 174 | 36 |
| SM(d33:2) | 0.001 | 0.008 | 0.02 | ns | ns | 23 | 8 | 12 | 9 | 20 | 7 |
| SM (34:2) | 0.001 | 0.01 | 0.0008 | ns | 0.04 | 26000 | 8081 | 17683 | 6874 | 23313 | 4800 |
| SM(d34:2) | 0.01 | 0.04 | 0.008 | ns | ns | 119 | 25 | 95 | 21 | 105 | 15 |
| SM (36:1) | 0.002 | 0.01 | 0.005 | ns | 0.004 | 12494 | 2723 | 9240 | 1815 | 12567 | 3656 |
| SM (36:2) | 0.00004 | 0.002 | 0.0002 | ns | 0.0006 | 16082 | 9067 | 8762 | 2857 | 14860 | 4022 |
| SM (d18:0/16:1(OH)) | 0.003 | 0.02 | 0.002 | ns | ns | 135 | 22 | 103 | 32 | 129 | 24 |

*- FDR corrected p-values.

Table S3. Metabolites significant in comparison V.

| metabolite | p-value | | | | | ALMS/BBS | | Ctrl | | Obese | |
| --- | --- | --- | --- | --- | --- | --- | --- | --- | --- | --- | --- |
|  |  |  |  |  |  | Intensity [counts x 10^3^] | | | | | |
|  | [ALMS/BBS] vs [Ctrl] vs [Obese] | [ALMS/BBS] vs [Ctrl] vs [Obese]* | [Ctrl] vs [ALMS/BBS] | [Obese] vs [ALMS/BBS] | [Obese] vs [Ctrl] | median | IQR | median | IQR | median | IQR |
| CAR 11:1 | 0.003 | 0.04 | ns | 0.0001 | 0.0002 | 213 | 102 | 132 | 68 | 101 | 76 |
| FA 26:1;O2 | 0.000008 | 0.0005 | 0.0001 | 0.0001 | ns | 20 | 26 | 59 | 19 | 68 | 15 |
| SPBP 18:1;O2 | 0.002 | 0.02 | 0.002 | ns | 0.02 | 280 | 51 | 200 | 141 | 251 | 77 |
| Sphingosine-1-phosphate | 0.0008 | 0.02 | 0.001 | ns | 0.007 | 532 | 115 | 346 | 273 | 485 | 138 |
| ST21:1;O2;S | 0.005 | 0.04 | 0.004 | ns | ns | 265 | 253 | 606 | 565 | 426 | 299 |
| LPC (18:2) | 0.0006 | 0.01 | 0.005 | ns | 0.001 | 34501 | 8749 | 45262 | 10701 | 32069 | 7276 |
| LPC (20:0) | 0.003 | 0.04 | 0.0002 | 0.03 | ns | 93 | 41 | 130 | 30 | 87 | 26 |
| LPC (20:1) | 0.002 | 0.03 | 0.02 | ns | 0.002 | 249 | 93 | 318 | 67 | 210 | 57 |
| LPC (24:0) | 0.002 | 0.03 | ns | 0.05 | 0.005 | 46 | 20 | 62 | 25 | 43 | 18 |
| LPC (O-18:1) /LPC (P-18:O) | 0.003 | 0.04 | ns | 0.0002 | 0.0001 | 75 | 68 | 108 | 26 | 60 | 18 |
| LPC(19:0) | 0.004 | 0.03 | ns | ns | 0.01 | 20 | 17 | 29 | 8 | 16 | 18 |
| LPC(20:0) | 0.005 | 0.04 | 0.001 | ns | 0.0004 | 28 | 10 | 38 | 10 | 25 | 10 |
| LPC(20:1) | 0.001 | 0.02 | 0.02 | ns | 0.001 | 84 | 27 | 107 | 24 | 73 | 19 |
| LPC(O-18:1) /LPC(P-18:0) | 0.0001 | 0.003 | 0.04 | ns | 0.01 | 26 | 20 | 37 | 12 | 18 | 4 |
| LPC(O-18:2) /LPC(P-18:1) | 0.007 | 0.05 | ns | 0.0004 | 0.0001 | 27 | 17 | 27 | 4 | 16 | 17 |
| LPE (O-18:1) /LPE (P-18:0) | 0.00005 | 0.002 | ns | 0.0001 | 0.01 | 353 | 198 | 253 | 147 | 172 | 32 |
| LPE (P-16:0) | 0.00002 | 0.0009 | 0.002 | 0.0001 | ns | 318 | 168 | 180 | 92 | 139 | 34 |
| LPE(O-18:0) | 0.006 | 0.04 | ns | ns | ns | 28 | 22 | 25 | 7 | 20 | 5 |
| LPE(P-16:0) /LPE(O-16:1) | 0.00004 | 0.001 | 0.01 | 0.0001 | ns | 192 | 91 | 130 | 67 | 108 | 19 |
| LPE(P-18:0) /LPE(O-18:1) | 0.00001 | 0.0006 | ns | 0.0001 | 0.004 | 204 | 110 | 158 | 95 | 104 | 30 |
| LPIM2 (19:0) | 0.00003 | 0.001 | ns | ns | ns | 25 | 11 | 11 | 12 | 17 | 8 |
| PC(16:0/20:4(OH)) | <0.000001 | <0.000001 | 0.0001 | 0.0001 | - | 1185 | 1225 | Not detected | - | Detected in 1 sample | - |
| PC(16:0/20:4(OH)) | <0.000001 | <0.000001 | 0.0001 | 0.0001 | - | 611 | 742 | Not detected | - | Detected in 2 samples | - |
| PC(18:0/18:2(OH)) | <0.000001 | <0.000001 | 0.0001 | 0.0001 | - | 362 | 1071 | Not detected | - | Detected in 1 sample | - |
| PC (36:3) | 0.0007 | 0.01 | 0.0006 | ns | ns | 33250 | 10186 | 39181 | 5422 | 36734 | 4353 |
| PC (38:7) | 0.002 | 0.03 | 0.0001 | 0.001 | ns | 76 | 85 | 122 | 50 | 83 | 57 |
| PC(18:2/18:2) | 0.0008 | 0.01 | ns | ns | ns | 948 | 775 | 1460 | 255 | 1137 | 501 |
| PC(18:2/20:4) | 0.0006 | 0.009 | 0.0001 | 0.0001 | ns | 269 | 136 | 472 | 85 | 376 | 72 |
| PC(18:3/18:2) | 0.005 | 0.04 | 0.0001 | 0.01 | ns | 42 | 36 | 78 | 36 | 49 | 22 |
| PC(36:4) | 0.0003 | 0.005 | ns | ns | ns | 101 | 101 | 179 | 35 | 135 | 48 |
| PE(18:2/P-16:0)/ PE(18:2/O-16:1) | 0.0003 | 0.005 | ns | 0.0001 | 0.0001 | 50 | 20 | 64 | 13 | 25 | 17 |
| PE(20:4/P-18:1)/ PE(20:4/O-18:2) | 0.002 | 0.02 | 0.0004 | ns | ns | 202 | 54 | 233 | 92 | 151 | 51 |
| SM (d18:2/14:0) | 0.004 | 0.04 | ns | 0.0008 | ns | 340 | 170 | 179 | 113 | 240 | 96 |
| SM(d33:2) | 0.006 | 0.04 | 0.0005 | 0.0007 | ns | 27 | 15 | 17 | 17 | 16 | 19 |

*- FDR corrected p-values.

*References:*

1. Marcinkiewicz-Siemion M, Ciborowski M, Ptaszynska-Kopczynska K, et al (2018) LC–MS-based serum fingerprinting reveals significant dysregulation of phospholipids in chronic heart failure. J Pharm Biomed Anal 154:354–363 . doi: 10.1016/j.jpba.2018.03.027

2. Gil de la Fuente A, Godzien J, Fernández López M, et al (2018) Knowledge-based metabolite annotation tool: CEU Mass Mediator. J Pharm Biomed Anal 154:138–149 . doi: 10.1016/j.jpba.2018.02.046

3. Godzien J, Ciborowski M, Martínez-Alcázar MP, et al (2015) Rapid and Reliable Identification of Phospholipids for Untargeted Metabolomics with LC–ESI–QTOF–MS/MS. J Proteome Res 14:3204–3216 . doi: 10.1021/acs.jproteome.5b00169

4. Ciborowski M, Teul J, Martin-Ventura JL, et al (2012) Metabolomics with LC-QTOF-MS Permits the Prediction of Disease Stage in Aortic Abdominal Aneurysm Based on Plasma Metabolic Fingerprint. PLoS One 7:e31982 . doi: 10.1371/journal.pone.0031982

5. Gil de la Fuente A, Traldi F, Siroka J, et al (2018) Characterization and annotation of oxidized glycerophosphocholines for non-targeted metabolomics with LC-QTOF-MS data. Anal Chim Acta 1037:358–368 . doi: 10.1016/j.aca.2018.08.005
